# *Thelazia callipaeda* in wild carnivores from Romania: new host and geographical records

**DOI:** 10.1186/s13071-016-1628-9

**Published:** 2016-06-18

**Authors:** Andrei Daniel Mihalca, Angela Monica Ionică, Gianluca D’Amico, Aikaterini Alexandra Daskalaki, Georgiana Deak, Ioana Adriana Matei, Vasile Șimonca, Daniel Iordache, David Modrý, Călin Mircea Gherman

**Affiliations:** Department of Parasitology and Parasitic Diseases, University of Agricultural Sciences and Veterinary Medicine of Cluj-Napoca, Calea Mănăștur 3-5, Cluj-Napoca, 400372 Romania; Department of Forest Mensuration and Wood Study, University of Agricultural Sciences and Veterinary Medicine Cluj-Napoca, Calea Mănăștur 3-5, Cluj-Napoca, 400372 Romania; Department of Game and Wildlife, Faculty of Silviculture and Forestry Engineering, Transilvania University, Şirul Beethoven 1, Brașov, 500123 Romania; Department of Pathology and Parasitology, University of Veterinary and Pharmaceutical Sciences, Palackého tř. 1946/1, Brno, 612 42 Czech Republic; CEITEC –VFU, University of Veterinary and Pharmaceutical Sciences, Palackého tř. 1946/1, Brno, 612 42 Czech Republic; Institute of Parasitology, Biology Centre of Czech Academy of Sciences, v.v.i., Branišovská 31, 370 05 České Budějovice, Czech Republic

**Keywords:** *Thelazia callipaeda*, *Canis aureus*, *Canis lupus*, *Felis silvestris*, Romania

## Abstract

**Background:**

*Thelazia callipaeda* is a vector-borne zoonotic nematode parasitizing the conjunctival sac of domestic and wild carnivores, rabbits and humans, with a vast distribution in Asia and the former Soviet Union. In Europe, the nematode has an emerging trend, being reported in Italy, France, Switzerland, Germany, Spain, Portugal, Bosnia and Herzegovina, Croatia, Romania, Greece and Serbia, with human cases known in Italy, France, Spain, Serbia and Croatia. In Romania, the infection was so far reported only in dogs, whereas there are no reports in wildlife despite the large numbers of wild carnivores in the country. The aim of this study was to evaluate the role of wild carnivores in the natural cycle of *T. callipaeda* in Romania.

**Methods:**

Between 2014 and 2016, 89 wild carnivores (64 golden jackals, *Canis aureus*, 13 grey wolves, *Canis lupus*, nine wildcats, *Felis silvestris* and three Eurasian lynxes, *Lynx lynx*) have been examined. During the necropsy, both eyes of all the examined animals have been thoroughly inspected for the presence of parasites. If present, all nematodes were collected in absolute ethanol (for molecular analysis of the partial *cox*1 gene) or in 4 % formalin (for morphological identification).

**Results:**

In total, three animals were found to be infected with *T. callipaeda*: a grey wolf, a golden jackal and a wildcat. The BLAST analysis of all the sequences showed a 100 % similarity to *T. callipaeda* haplotype h1. To the best of our knowledge, this study represents the first report of *T. callipaeda* in golden jackals, and the first study on *T. callipaeda* in wildlife from Romania.

**Conclusion:**

Our data broaden the host spectrum and geographical distribution of *T. callipaeda*, highlighting the role of wild carnivores as natural reservoirs for the infection and confirming the ongoing expanding trend of this zoonotic nematode in Europe.

## Background

*Thelazia callipaeda* is a vector-borne zoonotic nematode parasitizing the conjunctival sac of domestic and wild carnivores, rabbits and humans, with a vast distribution in Asia and the former Soviet Union. It is transmitted by drosophilid flies [1–4]. After the first report in Europe, in a dog from Italy [5], the infection has been found in various hosts in France, Switzerland, Germany, Spain, Portugal, Bosnia and Herzegovina, Croatia and Romania (as reviewed by Mihalca et al. [6]) and more recently in Greece [7] and Serbia [8]. Additionally, several human cases have been reported in Europe: from Italy and France [9], Spain [10], Serbia [8] and Croatia [11]. Despite its wide distribution and emerging trend, high local prevalence and zoonotic importance, most of the reports of *T. callipaeda* in Europe originate from domestic dogs and the number of records in European wild carnivores is relatively limited (Table 1).

**Table 1:** Reports of *Thelazia callipaeda* in wild carnivores from Europe

Host	Country	Reference
*Canis lupus*	Italy	[18, 23]
*Vulpes vulpes*	Italy	[18, 21, 26]
	Switzerland	[22]
	Portugal	[24]
	Bosnia and Herzegovina	[3]
*Felis silvestris*	Italy	[18]
*Martes foina*	Italy	[18]

In Romania, the infection was so far reported only in dogs [6, 12, 13] and despite the abundant wild carnivore populations [14], there are no reports of this nematode in wildlife. The aim of this study was to evaluate the role of wild carnivores in the natural cycle of *T. callipaeda* in Romania.

## Methods

**Fig. 1:**
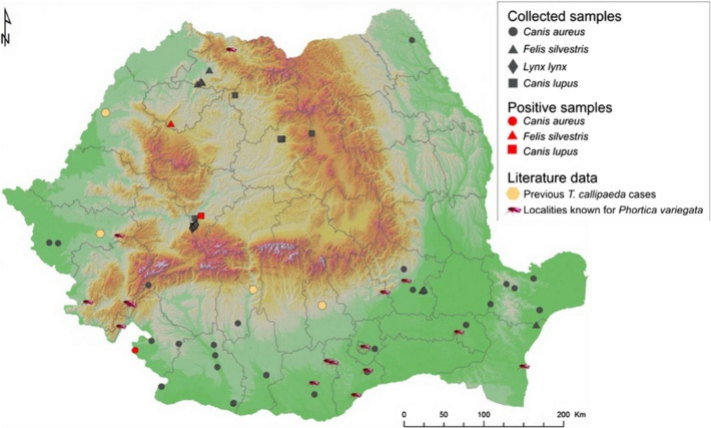
Geographical distribution of samples included in the study and the distribution of *Thelazia callipaeda* and its vector in Romania

Between 2014 and 2016, 89 large carnivores (64 golden jackals, *Canis aureus*; 13 grey wolves, *Canis lupus*; nine wildcats, *Felis silvestris*; and three Eurasian lynxes, *Lynx lynx*) were examined at the Department of Parasitology and Parasitic Diseases of the University of Agricultural Sciences and Veterinary Medicine of Cluj-Napoca, Romania (Table 2, Fig. 1). The animals originated from legal hunting or were road kills. In all animals a full parasitological examination was performed as part of a large scale collaborative project. During the necropsy, both eyes of all the examined animals were thoroughly examined for the presence of parasites. If present, all nematodes were collected either in absolute ethanol (for molecular analysis) or in 4 % formalin (for morphological identification). When the eye sockets were damaged, adjacent regions were also examined in order to reveal the possibly displaced parasites.

**Table 2:** Wild carnivores found positive for *Thelazia callipaeda*

Host	Total examined				Prevalence (%) (+/examined)^c^	Intensity (left/right eye)^d^	Locality (County)	Geographical coordinates
	M^a^	F^a^	M^b^	F^b^				
*Canis aureus*	13	13	18	20	1.6 (1/64)	6 M; 35 F/ 8 M; 21 F	Ostrovu Mare (Mehedinți)	44.3877342N, 22.5067806E
*Canis lupus*	0	0	9	4	7.7 (1/13)	1 M; 3F^e^	Pianu de Sus (Alba)	45.9242444N, 23.5055602E
*Felis silvestris*	3	0	3	3	11.1 (1/9)	2 F; 0 M/ 0 F; 0 M	Mesteacanu (Sălaj)	46.9624478N, 22.9832268E
*Lynx lynx*	0	0	2	1	0 (0/3)	–	–	–
Total					3.4 (3/89)	15 M; 61 F		

*M* males, *F* Females

^a^Juveniles (less than 1 year-old)

^b^Adults (more than 1 year old)

^c^All infected hosts were adult males

^d^ *M* males; *F* Females

^e^All nematodes were found in the nasal cavities, as the skull was significantly destroyed by the bullet and the parasites displaced from the typical location in conjunctival sac

For morphological examination, the nematodes collected in formalin were mounted on glass slides, cleared with lactophenol and examined using an Olympus BX61 microscope. Photographs and measurements for morphological identification were taken using a DP72 equipped with the Cell^^^F software (Olympus Corporation, Tokyo, Japan). Morphological identification was based on the features provided by Otranto et al. [15]. Genomic DNA was extracted from two nematodes from each positive animal (a total of six nematodes) using Isolate II Genomic DNA Kit (BIOLINE, London, UK) according to the manufacturer’s instructions. Amplification of a partial cytochrome *c* oxidase subunit 1 (*cox*1) gene of the nematodes (670 bp) was performed using the NTF/NTR primer pair, following reaction procedures and protocols described in [16]. PCR products were visualised by electrophoresis in a 2 % agarose gel stained with RedSafe™ 20,000× Nucleic Acid Staining Solution (Chembio, St Albans, UK) and their molecular weight was assessed by comparison to a molecular marker (O’GeneRuler™ 100 bp DNA Ladder, Thermo Fisher Scientific Inc., Waltham, MA,USA). Amplicons were purified using silica membrane spin columns (QIAquick PCR Purification Kit, Quiagen, Hilden, Germany) and sequenced at Macrogen Europe (Amsterdam, Netherlands). Sequences were compared to those available in GenBank^®^ dataset by Basic Local Alignment Search Tool (BLAST) analysis.

## Results

**Fig. 2:**
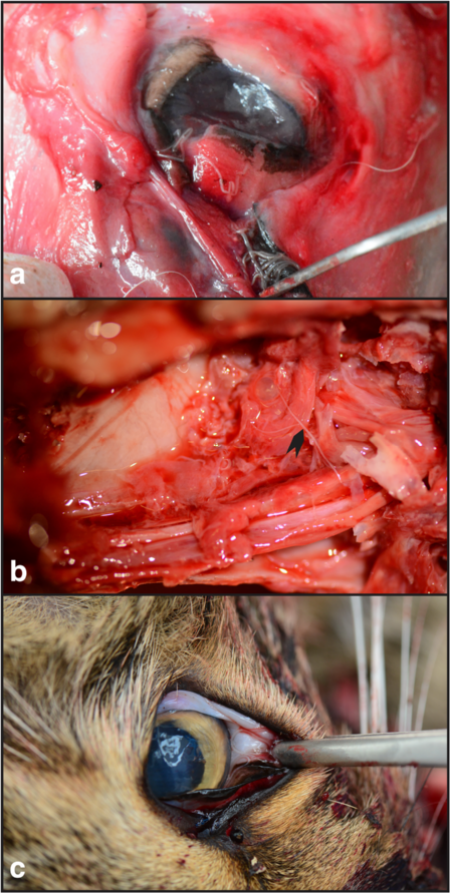
*Thelazia callipaeda* in wild carnivores from Romania: **a** in the conjunctival sac of a golden jackal (*Canis aureus*); **b** in the nasal cavities of a grey wolf (*Canis lupus*); the nematodes (*arrowhead*) have been accidentally displaced because of the extensive damage of the skull as the result of head injuries; and **c** in the conjunctival sac of a wildcat (*Felis silvestris*)

In total, three animals were found to host ocular nematodes: a grey wolf (*C. lupus*) (adult male), a golden jackal (*C. aureus*) (adult male) and a wildcat (*F. silvestris*) (adult male) (Table 2). In the golden jackal and in the wildcat, the nematodes were found in the conjunctival sac (Fig. 2a, c), while in the grey wolf, as the orbital area of the skull was severely damaged by the shooting trauma, the nematodes were found in the nasal cavities (Fig. 2b). All nematodes were identified morphologically as *T. callipaeda.* The BLAST analysis of all sequences obtained from the six molecularly investigated specimens showed a 100 % similarity to a sequence of *T. callipaeda* haplotype h1 (GenBank accession number AM042549) [17].

## Discussion

After the recent report of *T. callipaeda* in Romania in 2014 [6], two additional reports in dogs have suggested the spread of the disease to other areas of the country [12, 13]. So far, there are no confirmed human cases of infection with *T. callipaeda* in Romania. To the best of our knowledge, our findings represent the first reports of *T. callipaeda* in wild animals from Romania and, remarkably, the first report ever of this nematode in the golden jackal (*C. aureus*). This confirms the relatively low host specificity of *T. callipaeda*, as previously discussed by Otranto et al. [18], and the possible role of wild carnivores as natural reservoirs of infection [19]. The golden jackals, with an expanding range in Europe, have been recently shown to be important hosts also for *Dirofilaria* spp. in Romania [20].

Most of the data on *T. callipaeda* infecting wildlife in Europe refer to foxes, with a reported prevalence ranging between 5.1 % in northern Italy [21], 5.7 % in Switzerland [22], 27.7 % in Bosnia and Herzegovina [3] and 49.3 % in southern Italy [18]. In wolves, prevalence data are scarce and based on the examination of a few animals. In southern Italy, four out of five examined wolves were found infected [18, 23]. In wildcats from southern Italy, three out of eight examined animals were harbouring *T. callipaeda* [18]. Despite the lower frequency of the infection found in our study as compared to the data provided for wild carnivores in other countries, it seems that wild carnivores may deserve attention as possible natural reservoirs for *T. callipaeda* in Romania. However, their epidemiological role should be further investigated. The intensity of infection in wildlife from Europe is also highly variable. In foxes, the mean intensity varied between 3.8 nematodes per animal in Switzerland [22], 4.3 in northern Italy [21], 8.1 in Bosnia and Herzegovina [3] and 8.5 in Portugal [24]. In the previous reports of *T. callipaeda* from grey wolves, the intensity varied between 1 and 96 nematodes per animal [18, 23]. In our study, the highest intensity was found in the golden jackal (70 nematodes). As in most other studies on wildlife [18, 21, 24], our data show a sex ratio in favour of female nematodes.

The only confirmed vector for *T. callipaeda* is *Phortica variegata* (Diptera, Drosophilidae, Steganinae). It has been reported in Romania on various occasions [25] and its presence is known from the following counties: Buzău, Giurgiu, Constanța, Caraș-Severin, Mehedinți, Timiș, Maramureș, Ialomița and Teleorman. The low number of presence/absence studies on both the vector and the nematode may justify the incomplete overlap between the geographical distribution of the vector and the location of *T. callipaeda* cases found so far in Romania (as from Fig. 1).

## Conclusion

Our data broaden the known host spectrum and geographical distribution of *T. callipaeda*, highlighting the role of wild carnivores as possible natural reservoirs of infection and confirming the ongoing expanding trend of this zoonotic nematode in Europe.
